# Multimodule Human–Artificial Intelligence Collaboration Pipeline for Large Language Model–Assisted Thematic Analysis Across Digital Health Interview Studies: Comparative Evaluation Study

**DOI:** 10.2196/96129

**Published:** 2026-07-03

**Authors:** Yunbing Bai, Joseph Finkelstein

**Affiliations:** 1 Arizona Center for Telemedicine and Digital Health College of Medicine University of Arizona Tucson, AZ United States

**Keywords:** large language model, LLM, generative AI, thematic analysis, qualitative study, human-AI collaboration, health informatics

## Abstract

**Background:**

Qualitative thematic analysis is widely used in health research to examine patient experiences and inform the refinement of digital health interventions, but it is time- and labor-intensive. Large language models (LLMs) may help accelerate this process, yet their performance may depend not only on the model itself but also on how the analytic workflow is structured. Current evidence remains limited on how different LLMs perform across multistage thematic analysis workflows and across multiple health-related qualitative datasets.

**Objective:**

This study aimed to evaluate a modular human–artificial intelligence (AI) collaboration pipeline for LLM-assisted thematic analysis and compare how model choice and workflow strategy influence alignment between AI-generated and human-generated themes across 3 qualitative health studies.

**Methods:**

The framework was applied to analyze deidentified semistructured interview transcripts from 3 completed qualitative health studies involving patients with interstitial lung disease, postural orthostatic tachycardia syndrome, and chronic obstructive pulmonary disease. Three LLMs were compared: Gemini (Gemini 3 Pro), ChatGPT (GPT-5.2-thinking), and Opus (version 4.6). The workflow separated analysis into code extraction, code combination, and theme generation, and 5 strategies were tested. AI-generated themes were embedded using sentence-t5-xxl and compared with human-generated themes using cosine similarity after alignment with Hungarian and Greedy matching. Runtime and output-format consistency were also examined.

**Results:**

Output volume differed substantially by model. Gemini generated the fewest codes and themes, while ChatGPT showed a similar but higher output ceiling. Opus produced the largest and most variable codebooks and theme sets. Across the 3 studies, Opus showed the strongest and most consistent alignment with human-generated themes, with the best cosine similarity scores observed in postural orthostatic tachycardia syndrome–direct coding (mean 0.893, SD 0.041), chronic obstructive pulmonary disease–direct grouping (mean 0.891, SD 0.027), and interstitial lung disease–L3 (mean 0.889, SD 0.032). ChatGPT was competitive in selected settings, whereas Gemini generally produced slightly lower similarity scores but had the shortest runtime. ChatGPT and Opus also showed better formatting consistency and workflow usability than Gemini.

**Conclusions:**

A modular human-AI pipeline can support thematic analysis across multiple digital health interview studies, but performance depends strongly on both model choice and workflow design. Opus produced the most consistently human-aligned themes, while Gemini and ChatGPT showed different trade-offs in speed, fidelity, and usability. These findings support the use of LLMs as structured, human-supervised analytic assistants rather than replacements for qualitative researchers.

## Introduction

Qualitative methods are widely used in health research to examine patient experiences, identify barriers and facilitators to care, and guide the refinement of clinical and digital health interventions. Among these methods, thematic analysis is especially useful because it provides a flexible yet systematic approach for identifying, organizing, and interpreting patterns of meaning across textual data. Thematic analysis involves systematically identifying patterns in qualitative data through iterative coding and abstraction. For example, in a patient interview, a participant might state, “I get short of breath when walking even short distances, and it makes me anxious to leave the house.” A researcher may assign codes such as activity limitation*,* dyspnea*,* and anxiety for symptoms. Across multiple interviews, similar codes are grouped and refined into broader themes, such as the impact of symptoms on daily functioning or emotional responses to chronic illness. This process is inherently interpretive and requires balancing detail preservation with conceptual abstraction, making it both time-intensive and dependent on analyst judgment.

Foundational and health-oriented methodological literature emphasizes that rigorous thematic analysis depends on transparency, traceability, and a clear fit between analytic procedures and research aims [1-3]. In multidisciplinary health research teams, structured approaches such as the framework method can further improve consistency and auditability when working with large collections of interview transcripts. At the same time, qualitative analysis remains labor-intensive, requiring repeated transcript review, iterative code development, comparison across cases, and abstraction from codes to higher-level themes. These demands can make rigorous analysis difficult to scale, particularly in digital health research, where interview-based usability and implementation studies often need timely feedback to support intervention refinement. Prior studies of home telemanagement and rehabilitation technologies have further shown that understanding patient experiences, usability concerns, and practical barriers to participation is important for designing patient-centered systems for home use [4-7].

The use of artificial intelligence (AI) to support qualitative data analysis is not entirely new, but the recent emergence of large language models (LLMs) has substantially renewed interest in this area. Recent overviews show that AI-supported qualitative analysis has been applied across inductive and deductive coding, thematic analysis, discourse analysis, and analysis of large text corpora, suggesting that the current LLM wave should be understood as an acceleration of a longer methodological trajectory rather than an entirely new phenomenon [8,9]. What has changed is accessibility: contemporary LLMs can produce fluent codes, summaries, and theme descriptions directly from natural language prompts, making them attractive to researchers. However, this accessibility also raises the risk of superficial or poorly governed use if methodological safeguards are not clearly specified.

Recent literature on LLM-assisted qualitative analysis presents a mixed picture of promise and caution. Recent empirical and methodological studies have examined LLMs for qualitative coding, content analysis, grounded theory, thematic analysis, and thematic summarization in health and social science research [10-26]. These studies suggest that LLMs can support coding, theme generation, and synthesis, particularly when prompts are structured, and outputs are reviewed by human researchers [10-17,21-24,26]. For example, recent work has compared GPT-4 with human researchers in qualitative health care data analysis, evaluated LLMs for thematic summarization in qualitative health research, and tested ChatGPT-based protocols for thematic analysis and grounded theory [12,17,23,24]. At the same time, these studies consistently emphasize the need for human oversight, transparent prompts, validation procedures, and caution when interpreting AI-generated themes [8-12,18-22,25]. Taken together, the literature suggests that LLMs can contribute meaningfully to qualitative workflows, but their usefulness depends heavily on the dataset, task, model, prompt design, and workflow structure.

At the same time, more recent studies also highlight important limitations that are directly relevant to thematic analysis. Sakaguchi and colleagues [18] found that ChatGPT with GPT-4 achieved high agreement with human researchers for more descriptive themes in Japanese interview data, but its performance dropped markedly for themes requiring deeper cultural and emotional interpretation, with some agreement rates around 30%. Wen and colleagues [19] similarly showed that model choice matters: in a real-world charity-sector case study, GPT-4o achieved substantial agreement with human coders in multilabel categorization, whereas GPT-4o-mini showed only moderate agreement and greater instability across runs. These findings reinforce concerns that LLM outputs may be more reliable for surface-level pattern detection than for context-dependent interpretation, especially when cultural nuance, ambiguity, or subtle experiential distinctions are important. More critical methodological discussions have therefore argued that generative AI in qualitative data analysis introduces epistemic risks and should be treated as an assistive, human-supervised analytic tool rather than an autonomous interpreter [20].

Despite this growing literature, several gaps remain. First, many studies evaluate a single model, a single dataset, or a single prompting approach, which makes it difficult to separate model-specific behavior from workflow-specific effects. Second, much of the literature focuses on direct or near end-to-end prompting, with less attention to how multistage workflows may preserve or distort traceability as data move from transcripts to codes and from codes to themes. Third, evaluation practices remain heterogeneous: some studies emphasize feasibility, efficiency, or human comparison, whereas others compare thematic overlap or semantic similarity [13-17,23,25]. Few studies directly compare alternative workflow structures across multiple qualitative health datasets under standardized conditions. These gaps are especially important in health-related qualitative research, where less frequent but clinically meaningful concerns may be lost if models overcompress the data or privilege more common patterns [8,12,18-20,25].

Building on this literature, this study evaluates a modular human-AI collaboration framework for thematic analysis across 3 completed qualitative health studies involving patients with interstitial lung disease (ILD), postural orthostatic tachycardia syndrome (POTS), and chronic obstructive pulmonary disease (COPD) [27-29]. The framework decomposes thematic analysis into 3 discrete subtasks—code extraction, code combination, and theme generation—each conducted in independent LLM sessions to reduce context overload and improve traceability [30]. Using this framework, we compare 3 widely used LLM platforms (ChatGPT, Opus, and Gemini) and 5 workflow strategies, including both hierarchical and direct approaches to code consolidation and theme development [31-33]. AI-generated themes are then benchmarked against human-generated themes from the original studies using embedding-based semantic similarity and systematic alignment. By doing so, this study aims to clarify how model choice and workflow structure influence thematic fidelity, how iterative consolidation affects information retention, and what practical trade-offs emerge when implementing LLM-supported qualitative analysis in digital health research.

## Methods

### Model Selection

In this study, we selected 3 models from 3 widely used LLM platforms for evaluation and comparison. Specifically, we evaluated ChatGPT with GPT-5.2-thinking (with extended thinking; OpenAI), Opus (version 4.6; Anthropic), and Gemini (Gemini 3 Pro; Google) [31-33]. During the study window, extended thinking modes were available for ChatGPT and Opus but not for Gemini; therefore, each platform was evaluated using the relevant configuration available through its consumer-facing web interface at the time of analysis.

We intentionally used the consumer-facing web interfaces rather than the corresponding application programming interfaces (APIs), and each model-workflow condition was executed once using a standardized prompt and independent conversation, because the goal of this study was to evaluate a practical, researcher-facing workflow under conditions that resemble how many qualitative researchers currently interact with these tools in applied settings, rather than to benchmark API-level performance under fully programmatic control. This choice also allowed direct assessment of operational factors relevant to multistep qualitative analysis, including conversation organization, output exportability, and adherence to structured prompts within the native user interfaces.

All data collection and model interactions were conducted within a fixed study window from February 10, 2026, to March 30, 2026, to provide a temporal anchor for the model versions available at the time of evaluation. To reduce variability related to prior interaction history, all analyses were performed using newly registered accounts with paid personal subscriptions (ChatGPT Plus, Claude Pro, and Gemini Pro). Where available, settings that allow models to reference prior conversations were disabled (eg, “Reference chat history” in ChatGPT). Each analytical subtask was performed in a new, independent conversation so that no model could rely on prior session memory. The same standardized prompts, workflow logic, and transcript inputs were applied across all 3 platforms.

### Human-AI Collaboration Framework

**Figure 1:**
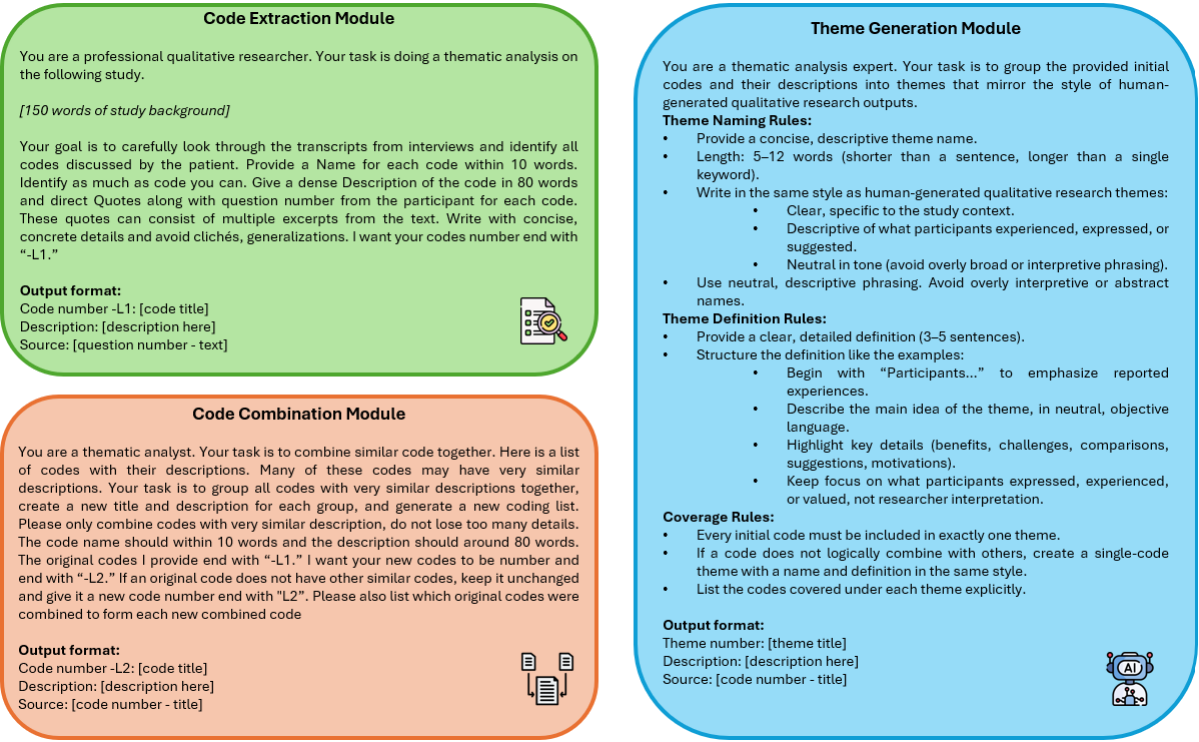
Verbatim standardized prompts used for the 3 modules in the modular human–artificial intelligence collaboration pipeline for large language model–assisted thematic analysis. This figure reproduces the exact prompt wording used across all analyses for the code extraction module, code combination module, and theme generation module. The prompts were applied consistently across ChatGPT (GPT-5.2-thinking), Opus (version 4.6), and Gemini (Gemini 3 Pro). The code extraction prompt instructs the model to identify transcript-level qualitative codes, assign concise titles, generate approximately 80-word descriptions, and cite question number–based supporting evidence. The code combination prompt instructs the model to merge semantically overlapping codes while preserving traceability to source code identifiers and standardized formatting. The theme generation prompt instructs the model to group codes into nonoverlapping, human-style qualitative themes with explicit rules for naming, definition structure, coverage, and source attribution.

To conduct thematic analysis, we applied a human-AI collaboration framework developed in our prior study [30]. In this framework, the overall analytical workflow is decomposed into a series of discrete, model-specific subtasks, each executed by a specialized LLM module. Each subtask is performed within an independent LLM conversation, and modules do not share memory or contextual knowledge across sessions. This modular architecture reduces per-module complexity, mitigates data loss caused by context-window limitations, lowers computational overhead, and improves the consistency and quality of the generated outputs. The framework consists of 3 specialized modules: a code extraction module, a code combination module, and a theme generation module. Figure 1 shows the prompts for the 3 modules.

The code extraction module is responsible for identifying all relevant qualitative codes from an input transcript. A standardized prompt is provided alongside the transcript, instructing the model to identify as many relevant codes as possible, assign each code a concise name limited to 10 words, and produce an approximately 80-word description for each code. The prompt also requires the model to cite the corresponding interview question number as supporting evidence, use clear and concrete language, avoid clichés or vague generalizations, and generate outputs in a predefined standardized format.

The code combination module consolidates overlapping or redundant codes in order to produce a more coherent and streamlined codebook. Using another standardized prompt, the model is instructed to group codes with substantially similar titles and descriptions and generate a new name and definition for each merged group. For codes without close counterparts, the original title and description are retained. To ensure traceability throughout the analysis process, the module records the original code number or numbers associated with each merged group, and the final results are produced in the same prescribed output format.

The theme generation module derives higher-level themes from the consolidated codebook. Its prompt directs the model to cluster codes and their descriptions into themes that reflect conventions commonly used in human-authored qualitative research. Detailed guidelines regarding length constraints, thematic structure, and stylistic requirements are embedded in the prompt to guide the generation process. Each code must be assigned to exactly 1 theme, ensuring complete coverage of the dataset without overlapping. The final output consists of a comprehensive list of themes, with each theme accompanied by a title, a descriptive summary, and the original code numbers associated with the codes it subsumes.

### Strategies for Using the Framework

**Figure 2:**
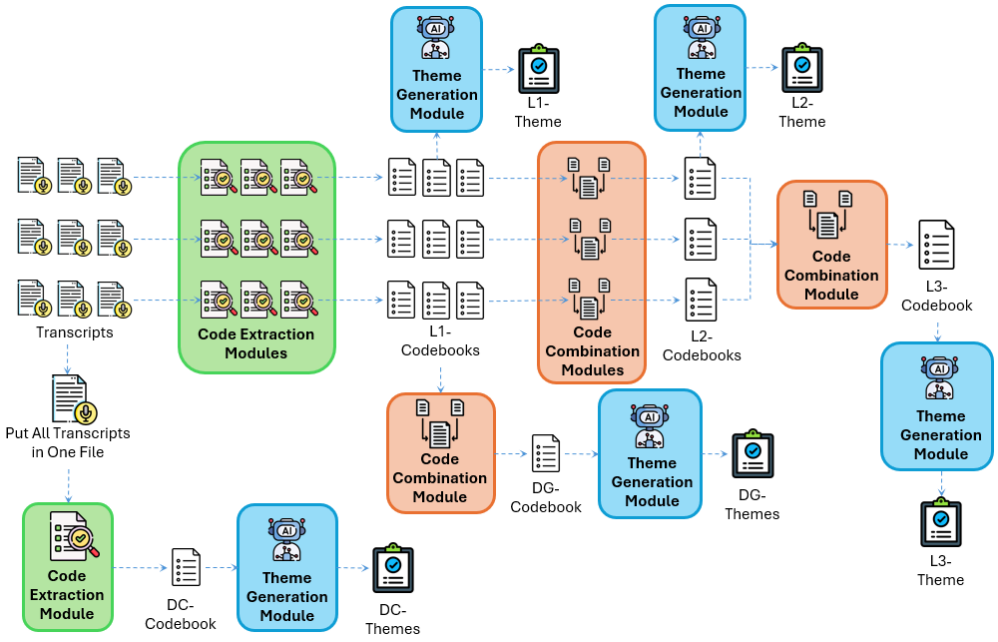
Overview of the multistage human–artificial intelligence collaboration pipeline and the 5 workflow strategies evaluated for large language model–assisted thematic analysis across different digital health qualitative interview studies. The framework was applied to deidentified semistructured interview transcripts, all drawn from previously completed telerehabilitation-related studies. The pipeline decomposes thematic analysis into 3 sequential large language model–assisted subtasks performed in independent conversations: code extraction, code combination, and theme generation. In the hierarchical workflows, transcripts are first analyzed individually to produce an L1 codebook, followed by iterative consolidation into an L2 and L3 codebook before theme generation. In the direct workflows, either all transcripts are analyzed together once to produce a DC codebook or the L1-codebook is submitted directly for 1-step consolidation to produce a DG codebook. Each resulting codebook (L1, L2, L3, DC, and DG) is then independently submitted to the theme generation module to produce a corresponding theme set. DC: direct coding; DG: direct grouping; L1: layer 1; L2: layer 2; L3: layer 3.

Figure 2 illustrates the workflow of the human-AI collaboration framework. The input for our framework would be a set of transcripts of interviews. Each transcript was uploaded as a .txt file into a new LLM conversation together with the standardized prompt for the code extraction module. Initiating a separate conversation for each transcript ensured that the interview context was preserved while respecting token-length constraints, maintained uniform analytical conditions, and prevented contamination from prior interactions.

After code extraction, one researcher (YB) collected the model-generated codes in text files. To reduce manual effort and the risk of identifier transcription errors, a short Python script (Python Software Foundation) was used solely to assign unique identifiers to the collected codes and generate 2 structured text files: a layer 1 (L1) codebook containing the code number, name, and definition for each code, and a quote-trace file containing the code number, code name, and corresponding interview question numbers. This deterministic organizational step did not modify code content, conduct qualitative analysis, or involve programmatic interaction with the LLM platforms, and it could be performed manually if needed.

To reduce redundancy, the code combination module was applied. The L1 codebook was divided into 4 subcodebooks to control the input length for each LLM conversation; each subcodebook contained the codes and descriptions derived from 4 to 5 transcripts. This partition was selected as a practical heuristic to balance input-length constraints and codebook diversity. Specifically, grouping 4-5 transcripts ensured that each subcodebook remained within a manageable size for the consumer-facing web interfaces used in this study, while still preserving sufficient variation in codes to support meaningful consolidation at the L2 stage. This approach allowed iterative merging without exceeding response length limits or overly compressing heterogeneous concepts in a single step.

These 4 subcodebooks were submitted independently to new LLM conversations together with the standardized code combination module prompt. The resulting outputs were then collected and processed to form the layer 2 (L2) codebook. To further consolidate overlapping codes, the L2 codebook was submitted again to the code combination module, producing the layer 3 (L3) codebook. Throughout this iterative consolidation process, parent-layer code numbers were preserved to maintain traceability between newly generated codes and their original source quotes.

In addition to this hierarchical workflow, we evaluated 2 straightforward strategies that did not impose input length controls. In the first approach, all transcripts were merged into a single txt file and submitted directly to the code extraction module, producing a direct coding (DC) codebook. In the second approach, the L1 codebook was submitted directly to the code combination module without dividing it into subcodebooks, producing a direct grouping (DG) codebook. Finally, all 5 resulting codebooks—L1, L2, L3, DG, and DC—were independently submitted to the theme generation module in separate LLM conversations using the same standardized prompt. The themes generated were then used for subsequent evaluation and comparison across the different analytical strategies.

### Semistructured Qualitative Interviews

The semistructured qualitative interviews were collected as part of 3 usability studies using a mixed methods design [27-29]. The studies aimed at assessing usability, attitudes, and acceptance of home-based telerehabilitation systems in patients with 1 of 3 conditions: COPD, POTS, or related dysautonomia, and ILD. All study participants were aged 18 years or older. They also needed the ability to complete study procedures, including usability testing and a semistructured interview. Individuals were excluded if they had medical conditions that could increase risk during the exercise‑related usability evaluation, including unstable angina, uncontrolled hypertension, a recent myocardial infarction, pacemaker use, painful or unstable bony metastases, recent skeletal fractures, or any other condition that, in the clinical or research team’s judgment, would make participation unsafe. Enrolled participants attended a single, 1‑hour, in‑person visit. After providing informed consent, research staff demonstrated the telerehabilitation system and supervised a practice session and then administered a sociodemographic questionnaire. Usability testing comprised 3 standardized tasks completed independently: logging into the system, completing a survey, and reviewing and performing an exercise routine. Analyses were stratified by the underlying medical conditions.

After usability testing, participants took part in a semistructured interview with open‑ended questions to explore their perceptions of the system and its potential for home‑based symptom management. Interviews were transcribed verbatim and deidentified. The qualitative interview data were analyzed using thematic analysis following Braun and Clarke’s [1] 6-phase process to organize, identify, and interpret key patterns and themes. The qualitative analysis team comprised a qualitative researcher (Aileen Gabriel) and a clinician-scientist (JF). The disagreements were resolved by a clinical informatician familiar with qualitative analysis. The iterative analysis continued until all codes were finalized and disagreements were reconciled. The research team repeatedly reviewed transcripts to immerse themselves in the data; initial codes were generated inductively through open coding, iteratively refined, and grouped into broader categories to capture patterns across participants. These categories were then synthesized into themes representing key aspects of participant experiences [27-29].

### Evaluation

For evaluation, we used the original deidentified interview transcripts and the final human-generated thematic analysis results from the 3 source studies. The ILD dataset included 17 transcripts, for which the human analysis generated 169 codes and 7 themes. The POTS dataset included 15 transcripts, for which the human analysis generated 178 codes and 10 themes. The COPD dataset included 16 transcripts, for which the human analysis generated 261 codes and 6 themes. The original transcripts were submitted as input to the LLM-assisted workflow, and the corresponding human-generated themes served as the reference standard for evaluating framework-derived themes.

All interview transcripts used in this study were deidentified and were not publicly available, published, or released before the present analysis. Therefore, direct exposure of the evaluated models to the source transcripts through publicly accessible training data is unlikely. The code extraction module prompt also included a study background field that was populated separately for each disease cohort. The exact verbatim text used for the ILD, POTS, and COPD datasets is provided in Multimedia Appendix 1.

**Figure 3:**
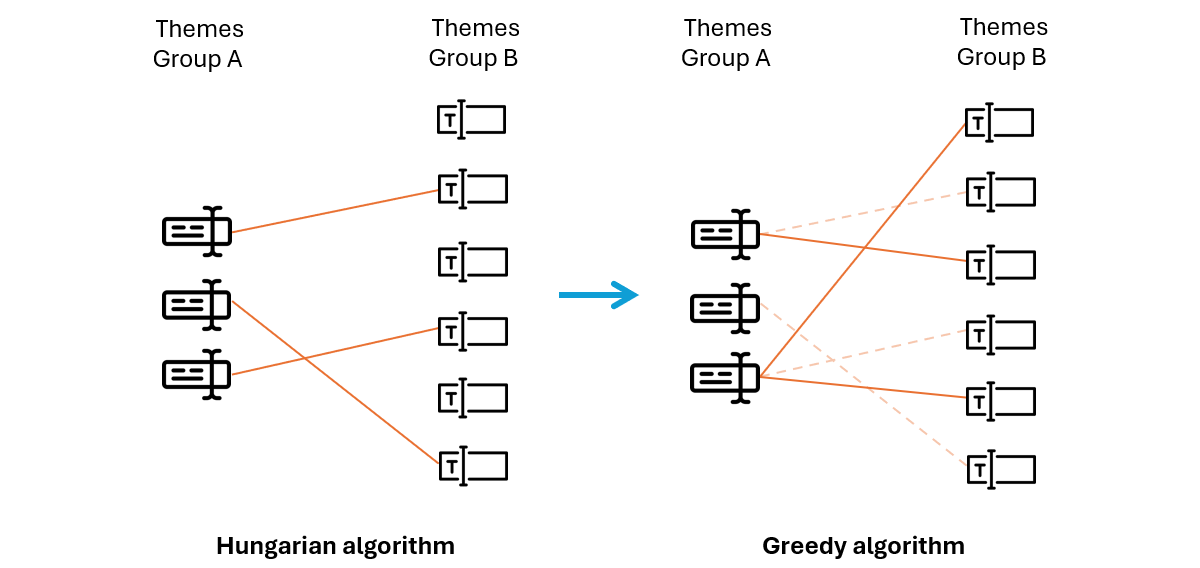
Themes alignment procedure is used to compare artificial intelligence (AI)–generated themes with human-generated reference themes across different qualitative health interview studies. This schematic illustrates the 2-step matching approach used after generating themes. First, theme descriptions from the human analysis and from each AI-generated output were embedded into 768-dimensional vector spaces using sentence-t5-xxl, and pairwise cosine similarity was calculated. Because the number of themes differed between the human and AI outputs across models and workflow strategies, the Hungarian algorithm was first applied to create a globally optimal one-to-one alignment between the smaller theme set and a subset of the larger theme set. Any remaining unmatched themes in the larger set were then assigned using a Greedy algorithm to the most similar theme in the smaller set. This combined procedure ensured that all themes were matched while maximizing overall semantic similarity.

To quantitatively assess the similarity between human-generated themes and those produced by the LLM, we used the sentence-transformer model sentence-t5-xxl, which has demonstrated strong performance on sentence similarity tasks, to embed the theme texts into a 768-dimensional dense vector space [34]. Because this model has a bounded maximum input length, we verified that all theme texts were below this limit; therefore, no truncation was applied during embedding. The resulting vectors were normalized to remove length bias and ensure that comparisons reflected semantic similarity rather than differences in text magnitude. Cosine similarity was then calculated for each pair of human and LLM theme vectors. Cosine similarity scores range from −1 to +1 and indicate the degree to which 2 texts describe conceptually similar content, with higher values representing greater semantic similarity. In addition, to verify traceability fidelity, we developed a Python script to validate whether each propagated parent-layer code identifier referenced an existing valid code in the immediately preceding codebook layer. The number of themes identified by humans and by the LLM often differed. To compare unequal theme sets while preserving both alignment quality and complete theme coverage, we used a 2-stage matching procedure combining the Hungarian algorithm and a Greedy nearest-neighbor assignment [35,36]. Figure 3 illustrates this alignment process.

First, the Hungarian algorithm was applied to the cosine similarity matrix to identify the globally optimal one-to-one alignment between the smaller theme set and an equally sized subset of the larger theme set, thereby establishing the primary bidirectional correspondences. Using the Hungarian algorithm alone would have excluded the remaining unmatched themes in the larger set from evaluation, which could bias the final similarity upward by omitting surplus themes with lower semantic similarity. Therefore, any remaining unmatched themes in the larger set were subsequently assigned to their most semantically similar theme in the smaller set using a Greedy nearest-neighbor procedure, ensuring that all themes contributed to the evaluation rather than discarding surplus themes. A Greedy-only strategy was not used because it does not enforce a globally optimal one-to-one primary alignment, and some themes in the opposite set may remain without any primary correspondence.

When multiple themes from the larger set mapped to the same theme in the smaller set, the cosine similarity values for those mappings were averaged rather than retaining only the single highest-scoring match. As a result, generating additional themes did not inherently improve the final score; extra weakly aligned themes could reduce the aggregate similarity. Basic statistical measures (average, standard deviation, maximum, median, and minimum) were then calculated for the resulting cosine similarity scores.

Processing time was measured manually using a digital clock or stopwatch. Timing began when the prompt and input file were submitted through the web interface and ended when the model completed its full response. Therefore, the recorded time represents end-user wall-clock processing time, including web interface latency, model response generation, and streaming time, rather than isolated model inference time. For theme generation tasks, processing time was measured 3 times, once for each study dataset. For L1 code extraction, 5 transcripts were randomly selected from the 48 available transcripts, and the corresponding extraction tasks were timed for each model. For the other tasks, processing time was measured across 5 randomly selected observations. Reported values are presented as mean (SD) in seconds.

We also conducted a brief descriptive assessment of output format consistency and workflow management. Format deviations were defined as responses that did not follow the prompt-specified structure and required manual correction before downstream processing, such as missing required fields, inconsistent numbering, omitted source or parent-code identifiers, or unrequested formatting changes. One researcher (YB) reviewed the generated outputs and recorded distinct format patterns. Workflow management was assessed descriptively based on whether the web interface supported project-level organization, easy tracking of multiple conversations, and convenient copying or exporting of outputs. Interrater agreement was not assessed because this was a practical workflow observation rather than a formal usability evaluation.

### Ethical Considerations

This study did not involve human participants or the collection of identifiable private information. No patient identifiers were accessed, recorded, or analyzed, and no attempt was made to reidentify individuals. In accordance with JMIR guidelines, this research does not meet the definition of human subjects research and was therefore exempt from institutional review board review and informed consent requirements. The study was conducted in compliance with applicable ethical standards for research using deidentified secondary data. The qualitative interview protocol was approved by the institutional review board of the University of Utah (protocol IRB_00168937).

## Results

For the AI-generated codes and themes, output volume varied substantially by strategy and model (Table 1). Gemini produced the fewest codes, with outputs remaining at approximately 10 codes per conversation, including definitions and traceability information, regardless of whether the task involved code extraction or code combination. ChatGPT showed a similar but higher capped pattern, producing approximately 25 codes per conversation across most tasks, except during the L2-to-L3 code combination step. In contrast, Opus did not exhibit a fixed-count pattern: extracted codes ranged from 15 to 60, and combined codes ranged from 43 to 153. These differences in code volume also shaped theme generation. Gemini produced fewer inputs for theme development and generated only 3-5 themes in several DC and DG settings, whereas Opus produced the largest codebooks and generally yielded the greatest number of themes. Python-based validation identified no invalid or hallucinated propagated code identifiers; all parent-layer code references were valid across models and consolidation stages.

**Table 1:** Counts of artificial intelligence–generated codes and themes across 3 large language models, 5 workflow strategies, and 3 digital health qualitative interview datasets^a^.

Category		ILD^b^			POTS^c^				COPD^d^		
		Gemini	ChatGPT	Opus	Gemini	ChatGPT	Opus	Gemini		ChatGPT	Opus
**AI^e^-generated codes, n**											
	L1^f^	185	314	395	155	303	341	159		295	357
	L2^g^	43	114	208	46	118	167	58		128	201
	L3^h^	15	64	129	13	52	85	13		78	114
	DC^i^	10	24	60	12	22	47	10		22	53
	DG^j^	10	28	153	13	25	91	29		50	107
**AI-generated themes, n**											
	L1	11	12	23	6	12	21	8		15	21
	L2	7	9	27	8	10	24	8		12	20
	L3	6	17	15	4	10	16	5		13	13
	DC	3	7	12	3	8	13	4		9	11
	DG	4	9	17	4	7	17	5		14	13

^a^This table presents the numbers of codes and final themes generated by Gemini, ChatGPT, and Opus when the modular human–artificial intelligence thematic analysis pipeline was applied to deidentified semistructured interview transcripts from patients with interstitial lung disease (n=17), postural orthostatic tachycardia syndrome or related dysautonomia (n=15), and chronic obstructive pulmonary disease (n=16). Results are shown for 5 workflow strategies: L1, in which themes were generated from transcript-level extracted codes; L2 and L3, in which themes were generated after successive code combination stages; direct coding, in which combined transcripts were submitted directly for code extraction; and direct grouping, in which the L1 codebook was submitted directly for code consolidation before theme generation.

^b^ILD: interstitial lung disease.

^c^POTS: postural orthostatic tachycardia syndrome.

^d^COPD: chronic obstructive pulmonary disease.

^e^AI: artificial intelligence.

^f^L1: layer 1.

^g^L2: layer 2.

^h^L3: layer 3.

^i^DC: direct coding.

^j^DG: direct grouping.

To examine whether these differences reflected broader output length behavior, we analyzed input and output word counts at each stage (Table 2). Input transcript lengths were comparable across models, but output lengths differed substantially. Gemini and ChatGPT produced relatively stable output lengths across tasks, with limited variation despite large differences in input size. This pattern suggests an effective output ceiling, where response length did not scale proportionally with input length. By contrast, Opus showed a more input-responsive pattern, with output length increasing in high-input settings such as DC and large codebook consolidation. This word count pattern supports the code and theme count findings, suggesting that Gemini and ChatGPT generated relatively bounded outputs, whereas Opus produced more variable and input-dependent results.

Input and output length also influenced the runtime. Table 3 summarizes end-user wall-clock processing time across models and workflow tasks. Gemini generally had the shortest processing time across most stages, while Opus required the longest time in code combination and theme generation tasks. The longer processing times for Opus are consistent with its larger and more variable outputs, which increased both response generation time and the amount of text carried into later workflow stages. Because processing time was measured through consumer-facing web interfaces, these values should be interpreted as practical workflow benchmarks rather than hardware-controlled inference-speed measurements.

**Table 2:** Input and output word counts across models and workflow stages in the modular human–artificial intelligence thematic analysis pipeline^a^.

Task	Gemini word counts, n				ChatGPT word counts, n				Opus word counts, n				
	Input		Output		Input		Output		Input		Output		
L1^b^ code extraction (n=48 transcripts)	1023 (277)		1174 (281)		1023 (277)		1946 (428)		1023 (277)		2128 (401)		
DC^c^ code extraction (n=3 datasets)	16,860 (1560)		1749 (232)		16,860 (1560)		2949 (154)		16,860 (1560)		8890 (391)		
L1-L2^d^ code combination (n=12 subcodebooks)	3119 (551)		1015 (157)		6180 (792)		2754 (731)		6302 (982)		3914 (793)		
L2-L3^e^ code combination (n=3 datasets)	3579 (345)		1164 (94)		10,310 (599)		5783 (797)		14,735 (1600)		8999 (1036)		
L1-DG^f^ code combination (n=3 datasets)	12,474 (1257)		1605 (457)		24,720 (842)		2876 (492)		25,210 (2482)		8502 (1711)		
L1 theme generation (n=3 datasets)	12,474 (1257)		1210 (258)		24,720 (842)		1800 (70)		25,210 (2482)		3118 (150)		
L2 theme generation (n=3 datasets)	3579 (345)		870 (113)		10,310 (599)		1617 (442)		14,735 (1600)		3063 (319)		
L3 theme generation (n=3 datasets)	1053 (101)		463 (81)		5479 (771)		1680 (148)		8506 (982)		1839 (192)		
DC theme generation (n=3 datasets)	807 (176)		376 (25)		1855 (87)		828 (26)		3747 (478)		1437 (77)		
DG theme generation (n=3 datasets)	3	1214 (500)		478 (21)		2404 (492)		958 (205)		7607 (1603)		2271 (167)	

^a^Values represent mean (SD) word counts for inputs and generated outputs at each stage for Gemini, ChatGPT, and Opus (Claude), applied to 3 qualitative health interview datasets (ILD, POTS, and COPD). Tasks include L1 code extraction, DC, hierarchical code combination (L1→L2, L2→L3), DG, and theme generation across codebook levels. Input lengths reflect submitted transcripts or codebooks, and outputs include generated codes, definitions, and traceability information. Gemini and ChatGPT showed relatively stable output lengths across tasks, whereas Opus exhibited more input-responsive output scaling.

^b^L1: layer 1.

^c^DC: direct coding.

^d^L2: layer 2.

^e^L3: layer 3.

^f^DG: direct grouping.

**Table 3:** Processing time across models and workflow tasks in the modular human–artificial intelligence thematic analysis pipeline^a^.

Task	Gemini (seconds), mean (SD)	ChatGPT (seconds), mean (SD)	Opus (seconds), mean (SD)
Extract codes from 1 transcript (L1^b^ extraction)	72 (6)	123 (16)	81 (9)
Combine codes from L1 codes in 4 transcripts (L1-L2^c^ combination)	91 (8)	185 (21)	273 (28)
Combine codes from L2 codes (L2-L3^d^ combination)	133 (11)	196 (19)	663 (56)
L1 theme generation	145 (13)	168 (16)	488 (42)
L2 theme generation	65 (5)	127 (17)	402 (36)
L3 theme generation	40 (4)	62 (5)	268 (25)

^a^Processing time was measured manually using a digital clock or stopwatch from prompt submission through the web interface to completion of the full model response. Theme generation tasks were measured 3 times, once for each study dataset, whereas all other tasks were measured across 5 observations. These values reflect end-user wall-clock time under consumer-facing web interface conditions and therefore include interface latency, response generation, and streaming time rather than isolated model inference time.

^b^L1: layer 1.

^c^L2: layer 2.

^d^L3: layer 3.

Table 4 shows the cosine similarity between human-generated themes and AI-generated themes under different settings, and Multimedia Appendix 2 visualizes the distribution of similarity scores. Because this study was designed as a descriptive workflow evaluation, the cosine similarity results are reported as descriptive summaries rather than inferential statistical comparisons. Across the 3 studies, Opus showed the strongest and most consistent alignment with human-generated themes, achieving the highest average cosine similarity in nearly all settings. Its scores were generally clustered around 0.88-0.89, with the best performance observed in POTS-DC (mean 0.893, SD 0.041), COPD-DG (mean 0.891, SD 0.027), and ILD-L3 (mean 0.889, SD 0.032). In comparison, ChatGPT showed competitive performance in some settings, particularly ILD-L1 (mean 0.870, SD 0.027), POTS-L2 (mean 0.881, SD 0.052), and COPD-DC (mean 0.872, SD 0.040), but its results were more variable across workflows. Gemini generally produced slightly lower similarity scores than the other 2 models, although it remained competitive in several direct-coding settings, such as ILD-DC (mean 0.866, SD 0.024) and COPD-DC (mean 0.865, SD 0.036). Word count of human-generated and AI-generated themes across models and workflow strategies was similar and lower than the limitation of the embedding models. Values are reported as mean word count with range and number of themes in Multimedia Appendix 3.

Across workflow strategies, no single strategy was universally best for all models, but some patterns were evident. For Opus, L3, DC, and DG consistently yielded the highest similarities, suggesting that either deeper hierarchical consolidation or DC strategies helped produce themes closer to human reference. For ChatGPT, performance was relatively stronger in L1 and L2, indicating that it may perform better with moderate structure rather than extensive consolidation. Gemini showed comparatively stable but less optimal performance, with its best results often appearing in DC or L1 settings. Overall, these findings suggest that model choice had a greater effect on theme similarity than workflow strategy, and that Opus combined with L3, DC, or DG produced the most human-aligned thematic outputs.

To help interpret the cosine similarity scores, Textbox 1 provides a representative example from the POTS dataset. The human-generated theme “biofeedback and self-monitoring” was closely matched with AI-generated themes from all 3 models, with cosine similarity scores of 0.89 for Gemini, 0.94 for ChatGPT, and 0.96 for Opus. These matched themes shared the central concept of using real-time physiological feedback, especially heart rate and oxygen saturation monitoring, to support safe exercise and symptom management. For comparison, the textbox also includes AI-generated themes that were mapped to different human themes in the full alignment but had lower cosine similarity scores when compared with “biofeedback and self-monitoring”. These examples show how scores decreased when the AI-generated theme focused on adjacent but less directly related concepts, such as home-based rehabilitation logistics, onboarding, device accessibility, or equipment constraints.

**Table 4:** Mean cosine similarity between human-generated themes and artificial intelligence–generated themes across 3 qualitative health interview studies, 5 workflow strategies, and 3 large language models^a^.

Study and themes			Gemini, mean (SD)		ChatGPT, mean (SD)		Opus, mean (SD)
ILD^b^							
	L1^c^	0.840 (0.024)		0.870 (0.027)		0.884 (0.029)	
	L2^d^	0.843 (0.027)		0.869 (0.032)		0.868 (0.034)	
	L3^e^	0.841 (0.035)		0.844 (0.042)		0.889 (0.032)^f^	
	DC^g^	0.866 (0.024)		0.851 (0.038)		0.882 (0.019)	
	DG^h^	0.848 (0.031)		0.865 (0.041)		0.882 (0.021)	
POTS^i^							
	L1	0.874 (0.046)		0.874 (0.038)		0.887 (0.039)	
	L2	0.856 (0.054)		0.881 (0.052)		0.888 (0.027)	
	L3	0.841 (0.021)		0.852 (0.050)		0.888 (0.035)	
	DC	0.859 (0.022)		0.872 (0.045)		0.893 (0.041)^f^	
	DG	0.859 (0.025)		0.866 (0.026)		0.882 (0.035)	
COPD^j^							
	L1	0.861 (0.039)		0.861 (0.041)		0.879 (0.041)	
	L2	0.861 (0.030)		0.852 (0.045)		0.884 (0.028)	
	L3	0.859 (0.042)		0.869 (0.038)		0.890 (0.031)	
	DC	0.865 (0.036)		0.872 (0.040)		0.888 (0.028)	
	DG	0.863 (0.037)		0.844 (0.038)		0.891 (0.027)^f^	

^a^This table summarizes semantic alignment between the original human-generated themes and artificial intelligence (AI)–generated themes produced by Gemini, ChatGPT, and Opus when the modular human-AI collaboration pipeline was applied to deidentified interview transcripts from 3 previously completed studies: ILD (17 patients evaluated during a 1-hour in-person feasibility session of a home-based telerehabilitation system, followed by semistructured interviews), POTS (15 patients with dysautonomia or POTS evaluated during a 1-hour in-person telerehabilitation usability session, followed by semistructured interviews), and COPD (16 patients interviewed remotely after completing a 12-month home-based pulmonary telerehabilitation program). Results are shown for 5 workflow strategies: L1, L2, L3, DC, and DG. Theme descriptions were embedded using sentence-t5-xxl, aligned using a combined Hungarian and Greedy matching procedure, and compared using cosine similarity. Higher values indicate greater semantic agreement between AI-generated and human-generated themes.

^b^ILD: interstitial lung disease.

^c^L1: layer 1.

^d^L2: layer 2.

^e^L3: layer 3.

^f^Highest values for each study.

^g^DC: direct coding.

^h^DG: direct grouping.

^i^POTS: postural orthostatic tachycardia syndrome.

^j^COPD: chronic obstructive pulmonary disease.

**A representative example illustrates how cosine similarity scores (CSSs) vary with thematic overlap between human-generated and AI-generated themes. This textbox presents 1 human-generated reference theme from the postural orthostatic tachycardia syndrome telerehabilitation dataset, “biofeedback and self-monitoring”, alongside artificial intelligence (AI)–generated themes from Gemini, ChatGPT, and Opus. The first row shows that the AI-generated theme matched to this human theme during the alignment procedure and its CSS. The second row shows an AI-generated theme from each model that was mapped to a different human theme in the full alignment, but was compared with “biofeedback and self-monitoring” to illustrate how similarity scores decrease when thematic overlap is partial or adjacent rather than direct. This example provides an interpretive anchor for understanding the cosine similarity range reported in the quantitative results.**

**Human theme**

- “Biofeedback and self-monitoring”: Real-time biofeedback, particularly heart rate and oxygen saturation monitoring, was viewed as essential for exercising safely with postural orthostatic tachycardia syndrome (POTS) symptoms. Participants reported that these features provided reassurance, guided exercise intensity, and helped prevent overexertion, enabling them to exercise independently with confidence. Visual displays, including engaging elements such as the racecar avatar, were appreciated for making monitoring intuitive and motivating. Tracking progress over time further reinforced adherence and a sense of accomplishment. Suggestions for improvement included making data more visible, adding instructional cues, expanding monitoring to blood pressure or symptom logging, and offering alerts to emergency contacts.

**Primary matched artificial intelligence–generated themes for this human theme**

- Gemini (CSS 0.89): Using Biofeedback and Structure for Safety and Symptom Management: Participants identified real-time physiological biofeedback, particularly heart rate monitoring, as a critical safety tool that transformed abstract sensations into actionable data for self-regulation. The structured nature of the program provided a verified medical framework that alleviated anxiety about overexertion, allowing users to move from avoidance behaviors to active management with confidence. Ultimately, users viewed this combination of biofeedback and guided pacing as a vital strategy for preventing “boom and bust” cycles and managing long-term stamina within the constraints of their condition.
- ChatGPT (CSS 0.94): Biofeedback-led pacing and safeguards address fear of flares: Participants described real-time vitals feedback (especially heart rate and oxygen) as central for pacing and safety during POTS-appropriate exercise. Participants reported using biofeedback to adjust intensity moment-to-moment and to decide when to slow down or stop, and some wanted additional vitals (eg, blood pressure) integrated to match their personal thresholds. Participants also wanted vitals displayed more prominently so that they can check quickly during exertion without competing visual elements. Participants linked these features to concerns about overexertion, symptom worsening, and the need for safeguards that respect “bad day” limitations and flexible intensity.
- Opus (CSS 0.96): Real-Time Physiological Monitoring for Safe Exercise Management: Participants with POTS identified continuous heart rate and oxygen saturation monitoring as medically essential for exercising safely, given the unpredictable cardiovascular responses characteristic of the condition. Real-time biofeedback allowed users to adjust exertion levels and avoid symptom flares from overexertion. Specific design feedback included repositioning the vital signs display for better visibility during active pedaling. Participants also requested the addition of blood pressure monitoring to provide a more complete physiological picture for both self-management and provider review.

**Comparison with artificial intelligence–generated themes mapped to another human theme**

- Gemini (CSS 0.80): Motivation, Accountability, and Logistical Factors in Home-Based Rehabilitation: Participants emphasized that the home-based model eliminated draining commutes, thereby conserving energy for exercise and democratizing access to care for those with mobility limitations. Engagement was further driven by gamified elements that provided mental distraction from physical exertion, as well as the professional accountability of knowing that a health care provider was monitoring their progress remotely. However, while the convenience was a primary driver for adherence, some participants noted that practical constraints, such as the need for dedicated physical space and equipment setup, presented logistical barriers in smaller living environments.
- ChatGPT (CSS 0.75): Guided Onboarding and Practice Builds Independent System Use: Participants reported that an initial walk-through or practice session was needed to learn how to use the system correctly, even when the interface later felt intuitive. Participants described onboarding as helping them understand where to click, how to start sessions, and how to set up the tablet and connected peripherals. Participants also suggested that future users with less technology experience may need additional support such as tutorial videos or remote guidance to replicate setup and use at home.
- Opus (CSS 0.77): Device Accessibility, Equipment Constraints, and Adoption Barriers: Participants identified practical barriers that could limit system adoption across diverse users and living situations. Requests for smartphone and tablet compatibility reflected the preference for accessing the system on primary daily devices rather than a dedicated computer. Physical space requirements for the recumbent pedal device and associated equipment were seen as potentially prohibitive for those in small living spaces. Cost concerns were raised as a hypothetical barrier given existing medical expenses, and participants noted that individuals with limited technology experience—particularly older adults—would require enhanced onboarding and tutorial support.

Across the full pipeline, ChatGPT and Opus also demonstrated stronger usability and task management support. Both platforms provided project-level conversation grouping, which facilitated tracking and managing the multiple conversations required by the pipeline. Gemini lacked an equivalent project management function; although this did not create a major limitation in our experimental setup (which used a new account with a single project), it may become more consequential in larger or longer-term studies.

Finally, with explicit output format requirements in the prompts, ChatGPT and Opus followed the requested format reliably and could export outputs as .txt files without additional prompting or tuning. Gemini was less consistent in adhering to the specified format: across 48 code extraction conversations, it produced 12 distinct formats; across 15 code combination conversations, it produced 5 formats; and across 15 theme generation conversations, it produced 8 formats. This variability increased downstream preprocessing effort relative to the other 2 models.

## Discussion

### Principal Findings

This study evaluated a modular human–AI collaboration pipeline for LLM-assisted thematic analysis across 3 qualitative digital health interview datasets and 5 workflow strategies. Overall, the findings show that both model choice and workflow structure shaped thematic outputs, but model choice appeared to have a stronger influence on alignment with human-generated themes. Across ILD, POTS, and COPD, Opus produced the most consistently human-aligned themes, while ChatGPT performed competitively in selected settings, and Gemini showed more constrained but relatively stable performance. These findings suggest that LLM-assisted thematic analysis should not be treated as a single generic capability; rather, performance depends on how a model interacts with specific workflow designs for extraction, consolidation, and theme generation.

A key finding is that Gemini and ChatGPT exhibited constrained output behavior under the evaluated conditions. Both models produced relatively fixed numbers of codes across tasks (approximately 10 for Gemini and approximately 25 for ChatGPT), even when input size and task complexity varied. In qualitative analysis, this matters because thematic fidelity depends on adequately capturing the diversity of participant experiences. When outputs are effectively bounded, there is a risk of undercoding distinct ideas and underrepresenting less frequent but potentially important perspectives. This risk is particularly important in health-related interviews, where less frequent concerns may still be clinically meaningful [18-20].

The word count analysis provides additional evidence supporting this pattern. Despite substantial variation in input length across workflow stages, Gemini and ChatGPT produced outputs with relatively stable lengths, suggesting the presence of an effective output ceiling under the evaluated conditions. In contrast, Opus exhibited more input-responsive behavior, with output length increasing in higher-input settings such as DC and large codebook consolidation. This difference in output length responsiveness is consistent with the observed variation in code and theme counts across models.

At the same time, the findings show that workflow structure can partly compensate for model-specific limitations. For Opus, the hierarchical strategies performed especially well, with L3 producing the highest or near-highest similarity in 2 of the 3 studies, and strong performance was also observed in DC and DG settings. This pattern suggests that Opus was able to absorb longer inputs and iterative consolidation without substantial thematic drift. In practical terms, this is encouraging for larger qualitative projects: a staged workflow that first extracts detailed codes and then merges them iteratively may reduce redundancy while still preserving conceptual distinctions. The strong performance of DC and DG for Opus also indicates that the model can tolerate broader inputs, giving researchers flexibility to choose between hierarchical and direct strategies depending on dataset size, desired granularity, and time constraints.

For ChatGPT, a different pattern emerged. Its performance was relatively stronger in L1 and L2, with less consistent results after deeper consolidation. This suggests that ChatGPT may benefit from moderately structured intermediate representations but lose details when codes are repeatedly merged. One interpretation is that each consolidation step reduces input richness, and because ChatGPT also showed capped output behavior, repeated merging may amplify information loss. In this sense, ChatGPT appears well suited to workflows that preserve more of the original code diversity rather than heavily compressing it. For small- to medium-sized datasets, this may make L1 or L2 especially useful, whereas for larger datasets requiring multiple rounds of consolidation, the trade-off between manageability and thematic detail may become more pronounced.

Gemini showed the lowest overall similarity scores in most settings, but its results were not uniformly weak. In several direct-coding settings, it remained competitive, and its performance was relatively stable across studies. However, Gemini’s strong tendency to generate approximately the same number of codes regardless of input size suggests limited responsiveness to transcript complexity. This likely constrained downstream theme generation by reducing the range of available concepts before theming even began. From a practical perspective, Gemini may still be useful when the analytic goal is rapid, high-level summarization rather than detailed qualitative interpretation. Its short outputs and fast runtimes make it efficient, but that efficiency appears to come at the cost of granularity and formatting consistency.

The runtime findings reinforce this interpretation of model behavior. Across the pipeline, processing time was driven less by the nominal task type than by cumulative input and output length. Opus had the longest runtimes because it also generated the most extensive output, which then increased the size of the next-stage inputs. Gemini was consistently the fastest, reflecting its shorter and more compressed responses. This highlights an important trade-off in applied LLM-assisted qualitative research: the most detailed outputs may better preserve thematic nuance, but they also require more time and create heavier downstream processing burdens. Therefore, researchers need to balance fidelity, scalability, and efficiency when selecting a platform and workflow.

Another practical contribution of this study is that it evaluates not only thematic similarity but also the operational usability of the platforms for multistep qualitative work. ChatGPT and Opus more reliably followed the required output structure and supported easier management of multiple conversations through project-level organization. Gemini was less consistent in output format, which increased preprocessing effort. These differences may seem secondary to thematic quality, but they matter in real-world research settings. A workflow that requires dozens of extractions, combinations, and theme generation sessions depends on consistent formatting and manageable conversation organization. Thus, platform usability should be considered alongside analytic performance when choosing tools for LLM-assisted qualitative analysis.

### Comparison With Prior Work

Compared with prior literature, this study contributes a more explicitly workflow-centered evaluation. Recent studies have shown that LLMs can support qualitative coding, content analysis, grounded theory, and thematic summarization, including comparisons between AI-generated and human-generated qualitative analyses [13-17,23-25]. However, many prior studies rely on single-prompt, single-model, or near end-to-end approaches. These approaches can be efficient, but they make it harder to identify where information loss occurs and often provide less traceability across analytic stages. By contrast, our pipeline separates code extraction, code consolidation, and theme generation into independent, standardized steps. This structure makes the analytic process more auditable and allows direct comparison of hierarchical versus direct strategies, aligning with recent recommendations that LLM-supported qualitative analysis should emphasize transparency, reproducibility, validation, and human oversight [8,10-12,20,22,24-26].

This study also adds a reproducible quantitative evaluation layer by comparing AI-generated themes with human-generated themes through embedding-based semantic similarity and systematic alignment. Although semantic similarity cannot fully capture qualitative rigor, it provides a useful comparative signal across models, workflows, and datasets [6,8,20]. In this study, the use of 3 separate health interview datasets strengthens confidence that the observed patterns are not tied to a single sample alone. Across ILD, POTS, and COPD, Opus remained consistently strong, while the relative strengths and weaknesses of ChatGPT and Gemini followed broadly similar patterns. This cross-study consistency suggests that workflow-model interactions may be a meaningful area for future methodological research in LLM-assisted qualitative analysis.

### Limitations

Several limitations should be noted. First, all analyses were conducted through consumer-facing web interfaces rather than APIs. Although we used newly registered paid accounts, disabled memory settings where available, and restricted data collection to a fixed study window, web-based platforms may still be affected by unannounced model updates, interface-level output constraints, and uncontrolled generation settings. In addition, the available configurations differed across platforms: ChatGPT and Opus were evaluated with extended thinking modes, whereas Gemini did not offer a comparable extended thinking option during the study window and was evaluated in its available standard configuration. Accordingly, Gemini’s shorter outputs and generally lower similarity scores may reflect differences in the available model-interface configuration, in addition to differences in model behavior.

Second, the study used standardized prompts and a modular workflow, which improved consistency across models but may not reflect performance under alternative prompt designs or less structured qualitative analysis workflows. In addition, each model-workflow condition was executed once to mirror practical researcher-facing use, so within-model stochastic variation was not quantified.

Third, the evaluation relied on embedding-based cosine similarity and a 2-stage Hungarian plus Greedy alignment procedure. This approach allowed all themes to be included in the comparison, but it is not equivalent to a formal precision-recall framework and does not fully capture qualitative rigor, interpretive depth, or potential overgeneration effects.

Fourth, this study was descriptive rather than confirmatory. Formal inferential testing was not conducted because theme pair similarity scores were generated through an alignment procedure, were not fully independent, and came from only 3 datasets with 1 run per model-workflow condition. Therefore, differences across models and strategies should be interpreted as descriptive patterns rather than statistically confirmed effects.

Although all LLM interactions were performed through consumer-facing web interfaces, to reduce manual labor and the risk of human error, this implementation used a simple Python-assisted identifier assignment step to organize intermediate outputs, which requires intermediate programmatic data-processing skills.

Finally, the study evaluated only 3 LLMs and included a descriptive assessment of output format consistency and workflow management conducted by one researcher (YB). Future work should include additional proprietary and open-source models, repeated runs, independent workflow assessments, and prespecified statistical analyses across larger and more diverse qualitative datasets.

### Future Directions

Future work should validate this framework across larger and more diverse qualitative datasets, disease areas, and methodological traditions. Additional studies should include a broader range of proprietary and open-source LLMs, repeated runs for each model-workflow condition, and API-based implementations with controlled decoding parameters to better assess reproducibility and run-to-run variability. Evaluation should also extend beyond embedding-based semantic similarity by incorporating expert ratings of theme quality, code-level validation linking excerpts to assigned codes, human-human comparison baselines, and measures of thematic coverage, missed concepts, and overgeneration. Future research could further examine how prompt design, transcript partitioning strategies, and human intervention at different stages influence analytic fidelity. These efforts would help clarify which combinations of model choice, workflow design, and human oversight are most appropriate for different qualitative research goals.

### Conclusions

This study shows that a modular human-AI pipeline can support thematic analysis across multiple digital health interview studies, but its effectiveness depends strongly on both model choice and workflow design. Opus achieved the most consistent alignment with human-generated themes, while ChatGPT and Gemini showed distinct trade-offs in thematic fidelity, runtime, and usability. These results suggest that LLMs can serve as valuable assistants in qualitative research when embedded within structured, transparent, and human-supervised workflows. Rather than replacing qualitative researchers, LLMs may be most useful as configurable analytic partners whose strengths and limitations must be carefully matched to the goals and scale of the study.

## Appendix

Multimedia Appendix 1 Exact verbatim study background text inserted into the code extraction module prompt for each qualitative interview dataset. The table reports the full study-specific background text used to replace the “[100 words of study background]” placeholder in the standardized code extraction module prompt before transcript-level analysis. Separate text was used for the 3 previously completed telerehabilitation-related qualitative studies involving patients with interstitial lung disease, postural orthostatic tachycardia syndrome, and chronic obstructive pulmonary disease.

Multimedia Appendix 2 Distribution of cosine similarity scores between human-generated themes and artificial intelligence (AI)–generated themes across 3 large language models, 5 workflow strategies, and 3 digital health qualitative interview studies. This figure visualizes the distribution of semantic similarity scores obtained after aligning AI-generated themes to the human-generated reference themes from 3 previously completed thematic analyses involving patients with interstitial lung disease (n=17), postural orthostatic tachycardia syndrome (n=15), and chronic obstructive pulmonary disease (n=16). For each model (Gemini 3 Pro, GPT-5.2-thinking, and Opus 4.6) and each workflow strategy (layer 1 [L1], layer 2 [L2], layer 3 [L3], direct coding [DC], and direct grouping [DG]), theme descriptions were embedded using sentence-t5-xxl and compared using cosine similarity after Hungarian plus Greedy matching. Higher scores indicate closer semantic alignment between the AI-derived themes and the original human thematic analysis. Across the 3 studies, Opus 4.6 generally showed the highest and most consistent similarity distributions.

Multimedia Appendix 3 Word count distribution of human-generated and artificial intelligence (AI)–generated theme summaries across models and workflow strategies. Values are reported as mean word count with range (minimum–maximum) and number of themes (n). Human-generated themes (n=23) were derived from 3 qualitative health interview studies (interstitial lung disease, postural orthostatic tachycardia syndrome, and chronic obstructive pulmonary disease). AI-generated themes were produced by Gemini 3 Pro, ChatGPT with GPT-5.2-thinking, and Opus 4.6 across the 3 studies using 5 workflow strategies: layer 1 (L1), layer 2 (L2), layer 3 (L3), direct coding (DC), and direct grouping (DG).

## Abbreviations

AI: artificial intelligence
API: application programming interface
COPD: chronic obstructive pulmonary disease
DC: direct coding
DG: direct grouping
ILD: interstitial lung disease
L1: layer 1
L2: layer 2
L3: layer 3
LLM: large language model
POTS: postural orthostatic tachycardia syndrome

## Data Availability

The data in our study are available from the corresponding author on reasonable request. The code can be accessed at the Arizona Telemedicine Program [37].
