# Supplementary material for: Multimodule Human–Artificial Intelligence Collaboration Pipeline for Large Language Model–Assisted Thematic Analysis Across Digital Health Interview Studies: Comparative Evaluation Study
Source: JMIR Med Inform. 2026 Jul 3;14:e96129. doi: 10.2196/96129 (PMC13379696; doi:10.2196/96129)
Supplement: Multimedia Appendix 1 [file medinform_v14i1e96129_app1.docx]

**Multimedia Appendix 1**

**Exact verbatim study-background text inserted into the Code Extraction Module prompt for each qualitative interview dataset.** The table reports the full study-specific background text used to replace the “[100-words of study background]” placeholder in the standardized Code Extraction Module prompt before transcript-level analysis. Separate text was used for the three previously completed telerehabilitation-related qualitative studies involving patients with interstitial lung disease (ILD), postural orthostatic tachycardia syndrome (POTS), and chronic obstructive pulmonary disease (COPD).

| **ILD** | **POTS** | **COPD** |
| --- | --- | --- |
| The objective of this qualitative feasibility study was to examine the usability and acceptability of a home-based telerehabilitation system to support pulmonary rehabilitation among patients with interstitial lung disease (ILD). Adults with a confirmed diagnosis of ILD were recruited using purposive sampling and participated in a single one-hour, in-person study visit. During the visit, a trained researcher demonstrated the Home Automated Telemanagement (HAT) system, after which participants independently completed a series of standardized tasks, including logging into the system, completing a symptom survey, and performing a guided exercise session using a tablet-based interface with physiological monitoring; an optional arm-bike exercise was also available. Following task completion, participants conducted a heuristic usability evaluation and took part in a semi-structured interview exploring their experiences with the system’s content, interface, and overall process, as well as its potential clinical impact on symptom management. Qualitative data from interviews were analyzed using thematic analysis to identify key themes related to usability, acceptability, and perceived benefits of telerehabilitation for home-based pulmonary rehabilitation in ILD patients. | The objective of this qualitative feasibility study was to explore the usability and acceptability of a home-based physical telerehabilitation system designed to support exercise engagement among patients with dysautonomia, including postural orthostatic tachycardia syndrome (POTS). Adults with dysautonomia or POTS-related symptoms were recruited to participate in a single one-hour, in-person study visit. After providing informed consent, participants received a guided demonstration and hands-on practice with the Home Automated Telemanagement (HAT) system, which integrates a recumbent cycling device, multimodal exercise instructions, and real-time physiological biofeedback. Participants independently completed standardized usability tasks, including system login, survey completion, and a guided supine cycling exercise while interacting with a tablet-based virtual interface. Following system use, semi-structured qualitative interviews were conducted to elicit participants’ perceptions of system usability, accessibility, safety, and potential for supporting home-based symptom management. Interview transcripts were analyzed using inductive thematic analysis to identify key themes related to user experience, perceived benefits, and areas for improvement | The objective of this qualitative study was to examine patient experiences with a 12-month home-based pulmonary telerehabilitation program delivered through the Home Automated Telemanagement (HAT) system among individuals with chronic obstructive pulmonary disease (COPD). COPD patients who had recently received care for an acute exacerbation were enrolled and participated in a long-term pulmonary rehabilitation program supported by HAT, a web-based telerehabilitation platform designed to promote adherence to personalized exercise regimens through instructional videos, remote monitoring, symptom tracking, and structured guidance. Upon completion of the program, participants engaged in semi-structured qualitative interviews conducted remotely by trained researchers to explore their experiences using the HAT system, including its usability, accessibility, and impact on exercise routines, self-management, and confidence. Interview data were analyzed using inductive thematic analysis to identify key themes related to the perceived benefits and challenges of the HAT-enabled telerehabilitation program, providing insights to inform the development of patient-centered pulmonary telerehabilitation systems for COPD care. |
