# Supplementary material for: Multimodule Human–Artificial Intelligence Collaboration Pipeline for Large Language Model–Assisted Thematic Analysis Across Digital Health Interview Studies: Comparative Evaluation Study
Source: JMIR Med Inform. 2026 Jul 3;14:e96129. doi: 10.2196/96129 (PMC13379696; doi:10.2196/96129)

**Multimedia Appendix 2**

**Distribution of cosine similarity scores between human-generated themes and AI-generated themes across 3 large language models, 5 workflow strategies, and 3 digital health qualitative interview studies.** This figure visualizes the distribution of semantic similarity scores obtained after aligning AI-generated themes to the human-generated reference themes from 3 previously completed thematic analyses involving patients with ILD (n=17), POTS (n=15), and COPD (n=16). For each model (Gemini-3-Pro, ChatGPT-5.2-thinking, and Opus-4.6) and each workflow strategy (L1, L2, L3, DC, and DG), theme descriptions were embedded using sentence-t5-xxl and compared using cosine similarity after Hungarian plus greedy matching. Higher scores indicate closer semantic alignment between the AI-derived themes and the original human thematic analysis. Across the 3 studies, Opus-4.6 generally showed the highest and most consistent similarity distributions. Abbreviations: L1, layer 1; L2, layer 2; L3, layer 3; DC, direct coding; DG, direct grouping.


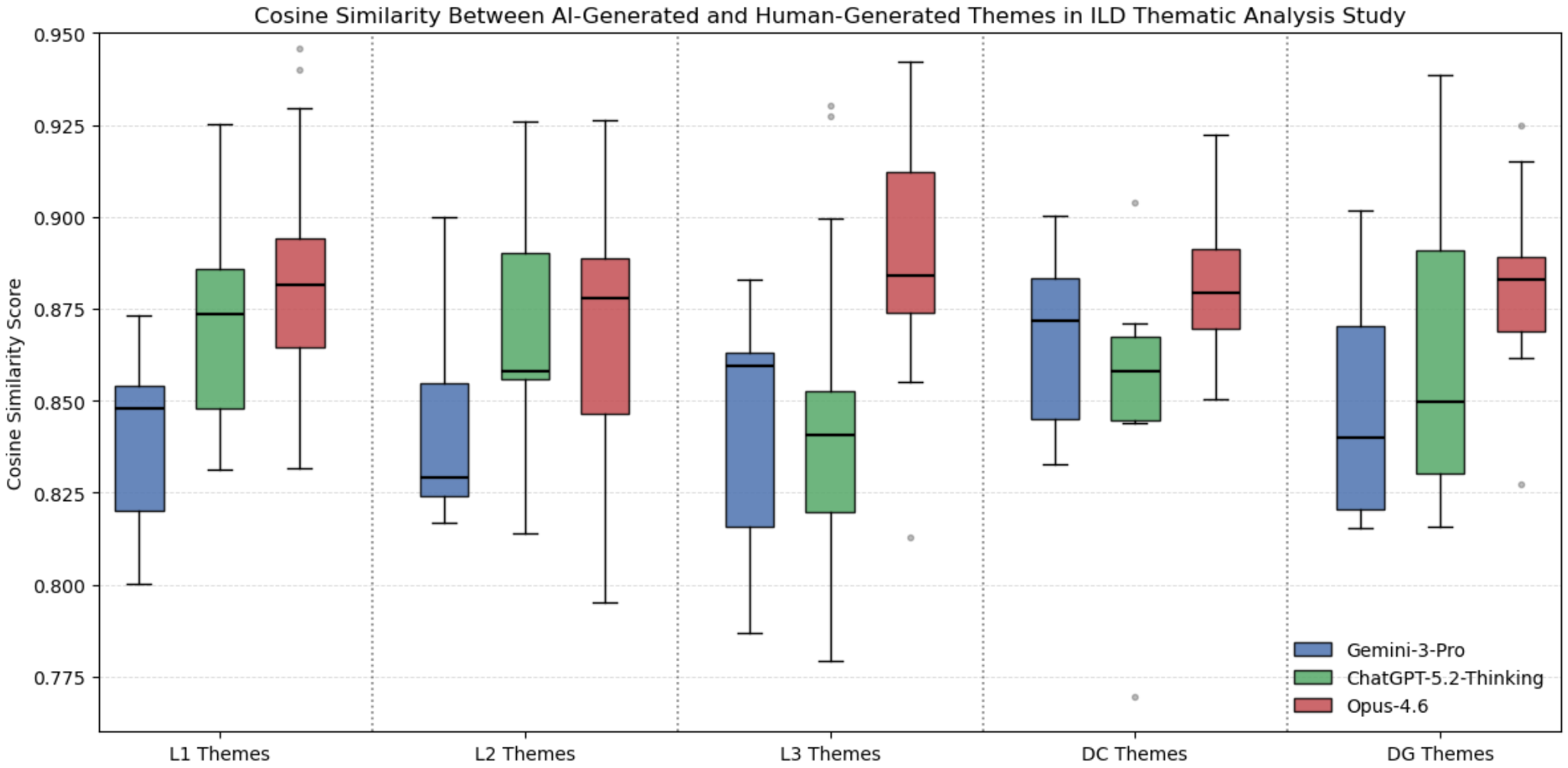


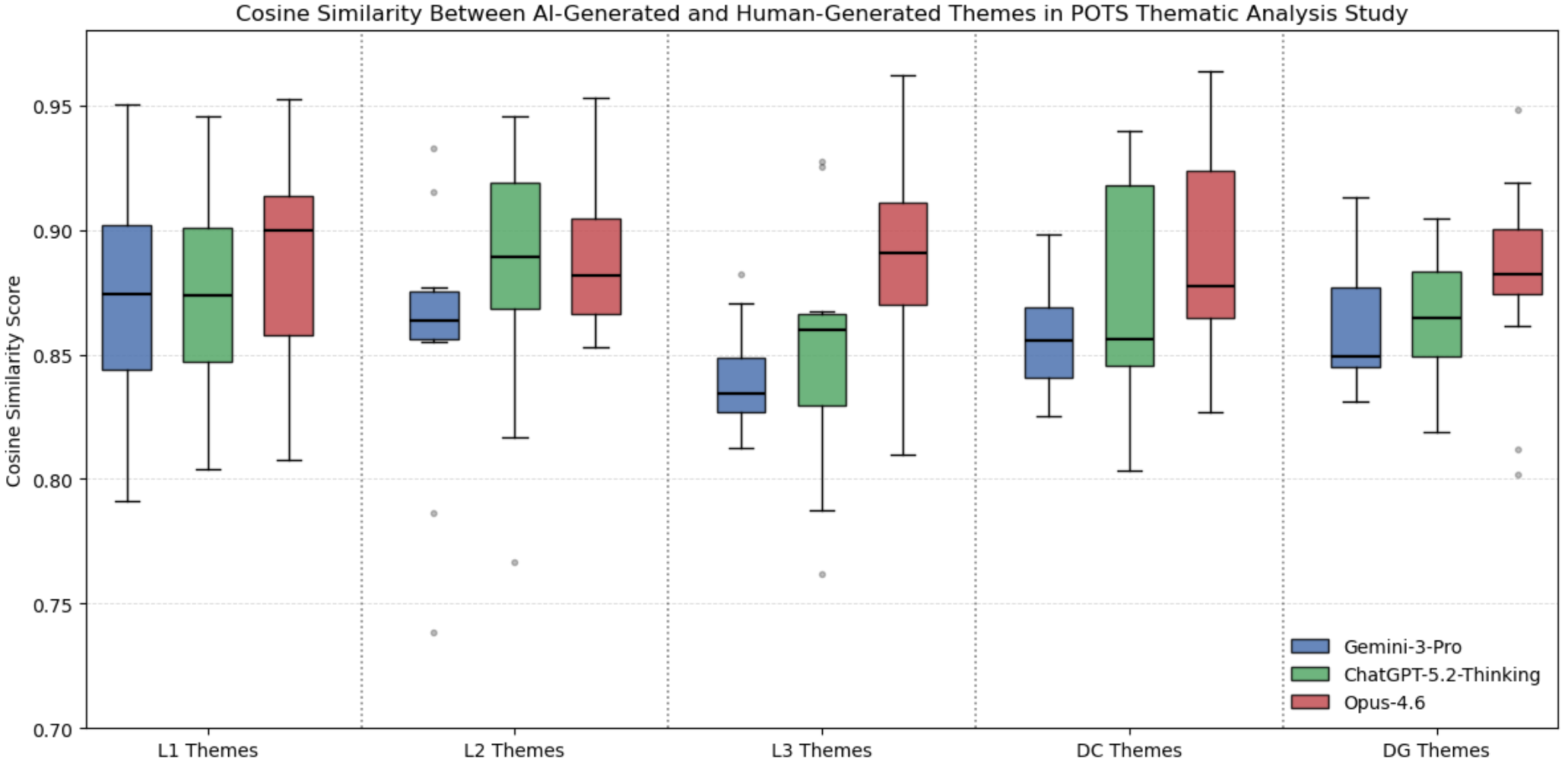


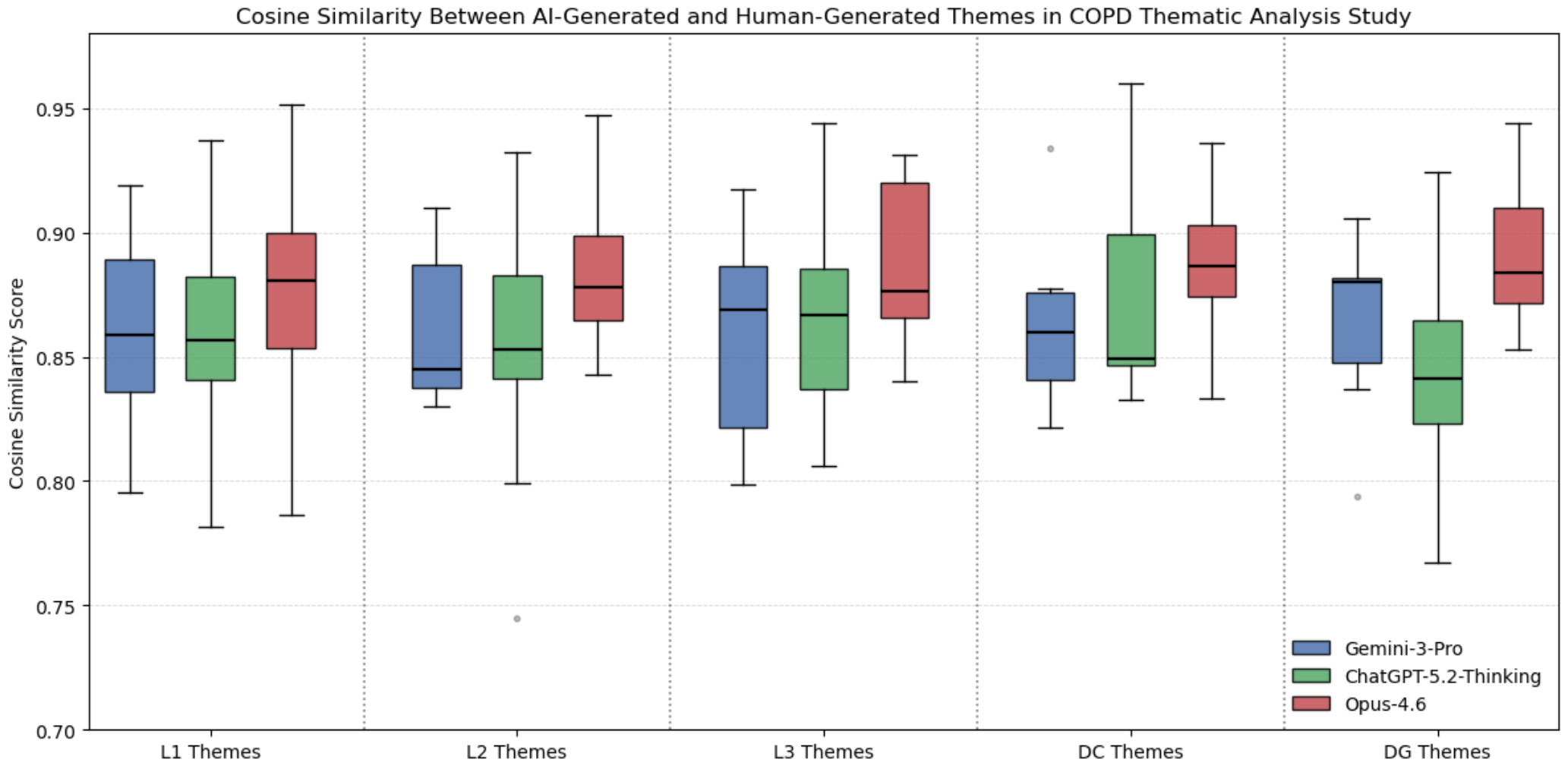

Supplement: Multimedia Appendix 2 [file medinform_v14i1e96129_app2.docx]
