# Supplementary material for: Multimodule Human–Artificial Intelligence Collaboration Pipeline for Large Language Model–Assisted Thematic Analysis Across Digital Health Interview Studies: Comparative Evaluation Study
Source: JMIR Med Inform. 2026 Jul 3;14:e96129. doi: 10.2196/96129 (PMC13379696; doi:10.2196/96129)
Supplement: Multimedia Appendix 3 [file medinform_v14i1e96129_app3.docx]

**Multimedia Appendix 3**

**Word count distribution of human-generated and AI-generated theme summaries across models and workflow strategies.** Values are reported as mean word count with range (minimum–maximum) and number of themes (n). Human-generated themes (n = 23) were derived from 3 qualitative health interview studies (ILD, POTS, and COPD). AI-generated themes were produced by Gemini-3-Pro, ChatGPT-5.2-thinking, and Opus-4.6 across the 3 studies using 5 workflow strategies: layer 1 (L1), layer 2 (L2), layer 3 (L3), direct coding (DC), and direct grouping (DG).

| **Average Word Count (words)** | | | |
| --- | --- | --- | --- |
| **Human Theme:** 94 (60 – 144), n = 23 | | | |
| **Codebook** | **Gemini-3-Pro** | **ChatGPT-5.2-Thinking** | **Opus-4.6** |
| L1-Theme | 88 (70 – 100), n = 25 | 96 (76 – 116), n = 39 | 106 (83 – 130), n = 65 |
| L2-Theme | 86 (59 – 99), n = 23 | 103 (80 – 149), n = 31 | 109 (84 – 131), n = 71 |
| L3-Theme | 77 (64 – 92), n = 15 | 91 (48 – 139), n = 40 | 106 (77 – 147), n = 44 |
| DC-Theme | 92 (82 – 109), n = 10 | 95 (76 – 132), n = 24 | 107 (80 – 153), n = 36 |
| DG-Theme | 86 (78 – 95), n = 13 | 86 (65 – 109), n = 30 | 110 (66 – 156), n = 47 |
